# Exit strategies: optimising feasible surveillance for detection, elimination, and ongoing prevention of COVID-19 community transmission

**DOI:** 10.1186/s12916-021-01934-5

**Published:** 2021-02-17

**Authors:** K. Lokuge, E. Banks, S. Davis, L. Roberts, T. Street, D. O’Donovan, G. Caleo, K. Glass

**Affiliations:** National Centre for Epidemiology and Population Health, Research School of Population Health, The Australian National University, 62 Mills Road, ACT 2601 Acton, Australia

**Keywords:** Community transmission chains, COVID-19, Detection, Modelling, Primary care, Surveillance, Syndromic fever, Testing

## Abstract

**Background:**

Following implementation of strong containment measures, several countries and regions have low detectable community transmission of COVID-19. We developed an efficient, rapid, and scalable surveillance strategy to detect remaining COVID-19 community cases through exhaustive identification of every active transmission chain. We identified measures to enable early detection and effective management of any reintroduction of transmission once containment measures are lifted to ensure strong containment measures do not require reinstatement.

**Methods:**

We compared efficiency and sensitivity to detect community transmission chains through testing of the following: hospital cases; fever, cough and/or ARI testing at community/primary care; and asymptomatic testing; using surveillance evaluation methods and mathematical modelling, varying testing capacities, reproductive number (R) and weekly cumulative incidence of COVID-19 and non-COVID-19 respiratory symptoms using data from Australia. We assessed system requirements to identify all transmission chains and follow up all cases and primary contacts within each chain, per million population.

**Results:**

Assuming 20% of cases are asymptomatic and 30% of symptomatic COVID-19 cases present for testing, with *R* = 2.2, a median of 14 unrecognised community cases (8 infectious) occur when a transmission chain is identified through hospital surveillance versus 7 unrecognised cases (4 infectious) through community-based surveillance. The 7 unrecognised community upstream cases are estimated to generate a further 55–77 primary contacts requiring follow-up. The unrecognised community cases rise to 10 if 50% of cases are asymptomatic. Screening asymptomatic community members cannot exhaustively identify all cases under any of the scenarios assessed. The most important determinant of testing requirements for symptomatic screening is levels of non-COVID-19 respiratory illness. If 4% of the community have respiratory symptoms, and 1% of those with symptoms have COVID-19, exhaustive symptomatic screening requires approximately 11,600 tests/million population using 1/4 pooling, with 98% of cases detected (2% missed), given 99.9% sensitivity. Even with a drop in sensitivity to 70%, pooling was more effective at detecting cases than individual testing under all scenarios examined.

**Conclusions:**

Screening all acute respiratory disease in the community, in combination with exhaustive and meticulous case and contact identification and management, enables appropriate early detection and elimination of COVID-19 community transmission. An important component is identification, testing, and management of all contacts, including upstream contacts (i.e. potential sources of infection for identified cases, and their related transmission chains). Pooling allows increased case detection when testing capacity is limited, even given reduced test sensitivity. Critical to the effectiveness of all aspects of surveillance is appropriate community engagement, messaging to optimise testing uptake and compliance with other measures.

**Supplementary Information:**

The online version contains supplementary material available at 10.1186/s12916-021-01934-5.

## Background

Worldwide, countries are implementing measures to contain the Coronavirus Disease 2019 (COVID-19) and a number of countries, including China, South Korea, Hong Kong, Singapore, Taiwan, Australia, Vietnam, and New Zealand have achieved low community transmission levels. All countries that have substantially controlled COVID-19 transmission have implemented initial strong containment and social distancing, combined with extensive surveillance [1–3]. For example, in Australia community compliance with social and movement restrictions, estimated at over 80% [4], along with strong border controls and wider testing and management of identified cases and contacts, has led to case numbers dropping from a peak of almost 700 cases reported per day during a resurgence of transmission in August to zero locally acquired cases for several days prior to 1 December 2020 [5].

Once strong containment, social distancing and other measures have successfully reduced case numbers, enhanced surveillance systems are needed to confirm disease elimination and detect and control any reintroductions of disease into the community. Previous modelling raised concerns that following successful COVID-19 control, there is likely to be a resurgence of transmission when measures are lifted that cannot be detected or managed through surveillance of hospital presentations [6].

Community-based surveillance and contact-tracing is a proven strategy for enabling early detection in infectious disease outbreaks [7] and has the potential to prevent resurgence. Testing capacity, as well as case and contact follow-up interventions, is continuing to improve internationally, providing more sensitive and sophisticated surveillance systems [8–10].

Currently, there is uncertainty on how best to identify all cases once weekly cumulative incidence is very low and to detect and manage any reintroduction of community transmission on an ongoing and sustainable basis. The World Health Organization (WHO), although recommending surveillance of influenza-like illness and severe respiratory illness, identifies these areas as a knowledge gap in the monitoring of COVID-19 community transmission [2]. This lack of evidence hampers effective disease management and contributes to a reluctance to implement strong containment measures, due to fears that they would be required for extended periods [11, 12]. These fears can be addressed by a clear strategy for effectively identifying and managing early re-emergence of community transmission once controls are lifted through surveillance and management of cases and contacts, without the need for reinstitution of widespread containment measures.

Exhaustive detection and elimination of community transmission chains, as recommended by WHO representatives [13], is standard practice in management of high-risk pathogen outbreaks such as Ebola virus disease [7, 14], and this practice provides useful guidance for the control of COVID-19 [1]. Although some factors make COVID-19 more difficult to control than Ebola virus disease (e.g. pre-symptomatic transmission, a higher proportion of asymptomatic disease, and shorter serial interval), it is likely more amenable to control than influenza due to the longer serial interval.

This study evaluates surveillance options and proposes an efficient, feasible and scalable strategy that will:

- Exhaustively identify transmission chains;
- Provide strong evidence that community transmission has been eliminated;
- Identify and manage any reintroductions of disease when control measures are lifted;
- Address constraints such as limited testing capacity; and
- Identify broader response priorities that are critical to enhanced surveillance.

A range of options are assessed, and efficient strategies recommended, with the structure and requirements for the system summarised. Given constraints on testing, we considered a range of testing requirements to allow the proposed strategy to be tailored to capacity and to consider the potential benefits of pooling [15–17].

## Methods

We considered the following three candidate groups for surveillance:

- Group 1: Patients with pneumonia presenting to hospitals
- Group 2: Patients presenting with acute respiratory symptoms (fever and cough and/or other acute respiratory illness symptoms such as runny nose, sore throat) for testing in the community (e.g. at primary care or specific respiratory/COVID clinics and testing facilities)
- Group 3: Asymptomatic community members, including those who may be at higher risk of unprotected exposure (e.g. supermarket and delivery workers, transport workers [18], essential service staff).

Fever and cough was the syndromic case definition recommended by WHO early in the outbreak [2], and although often reported in patients with COVID-19. It is clear that a substantial proportion of confirmed COVID-19 cases do not report these symptoms [20]. We therefore also considered a broader syndromic surveillance case definition such as that utilised in Australia since April 2020, which included any acute respiratory symptoms such as cough, sore throat, runny nose, cold symptoms or flu-like symptoms [21].

The following were estimated for the above three groups:

- The estimated number of cases in the community within the same transmission chain as the detected case, at the time the case is detected through the surveillance system.
- The sensitivity and efficiency of testing these groups for a range of testing capacities and varying weekly cumulative incidence of COVID-19 and non-COVID-19 respiratory symptoms in the population under surveillance. Efficiency was assessed by considering the number of tests required per case detected, and the number of cases missed, under different scenarios.
- The feasibility of surveillance and system requirements for increasing testing and follow-up.

Gains from enhanced community surveillance were estimated for two scenarios:

- Default transmission scenario, with limited or no containment measures and an assumed reproductive number of 2.2 when cases occur [22].
- Low transmission scenario, with some containment measures in place and therefore a reproductive number of 1.2

Estimated cases in the community were calculated using a stochastic susceptible-exposed-infectious-recovered (SEIR) model, assuming a 4-day incubation period before symptom onset, with transmission occurring during the final 2 days of this period. That is, a latent period of 2 days followed by 2 days of asymptomatic transmission in all infected individuals. We assumed three levels of severity: severe cases (20% of all cases) [23] who are hospitalised, asymptomatic cases (20% of all cases) [24], and the remaining 60% of cases experiencing mild or moderate disease and not requiring hospitalisation. In sensitivity analyses, we considered proportions of asymptomatic infection of either 20 or 50% [25]. As hospital surveillance will reflect cases with severe disease, we assumed a 7-day delay from symptom onset until presentation at hospital in severe cases [26], while a proportion of mild and moderate cases present for community testing 2 days after developing symptoms. We assume that cases are infectious for 4 days (including the 2 days of pre-symptomatic transmission described above) to replicate serial intervals of around 4–6 days [26]. Additional compartments in the model for individuals presenting to primary care and to hospital allowed us to identify the time until an event occurred, and the model was run until either the disease had died out or there had been a presentation at both primary care and the hospital. Repeated runs of the model allowed us to calculate averages over many stochastic simulations. Full model code is provided as a supplementary document (S1).

Sensitivity and efficiency of testing strategies was evaluated using the following assumptions:

- Random sampling of presentations in each group.
- Weekly cumulative incidence of the syndromic surveillance case definition ranging from 2 to 6% of the population under surveillance: this range was chosen based on several data sources, namely, in the absence of control measures 3% (peak), 2% (winter) and 1% (outside winter) fever and cough weekly cumulative incidence in line with Australian syndromic surveillance data (FluTracking) [27], and weekly acute respiratory symptoms (cold or flu-like symptoms as defined previously) incidence of 6% in winter (June) [28]. With social distancing measures, incidence of fever and cough has been shown to decrease by 40% [29], a finding also seen in Australian data [30].
- Testing rates of 30% [28], 50% and 100% in all patients with symptoms.
- Weekly cumulative incidence of COVID-19 ranging between 0.01 and 0.5%, based on reporting levels across high- and low-transmission settings with high test coverage [31].

We also assessed overall test performance (negative and positive predictive values and false negative and false positive rates) under a range of weekly cumulative incidence values, sensitivity and specificity. Benefits of pooling of samples were considered by estimating the number of tests required at a given weekly cumulative incidence. We assessed pooling by comparing a scenario with high test sensitivity and no pooling, to a scenario of pooling under reduced test sensitivities.

## Results

### Gains from early detection

**Fig. 1:**
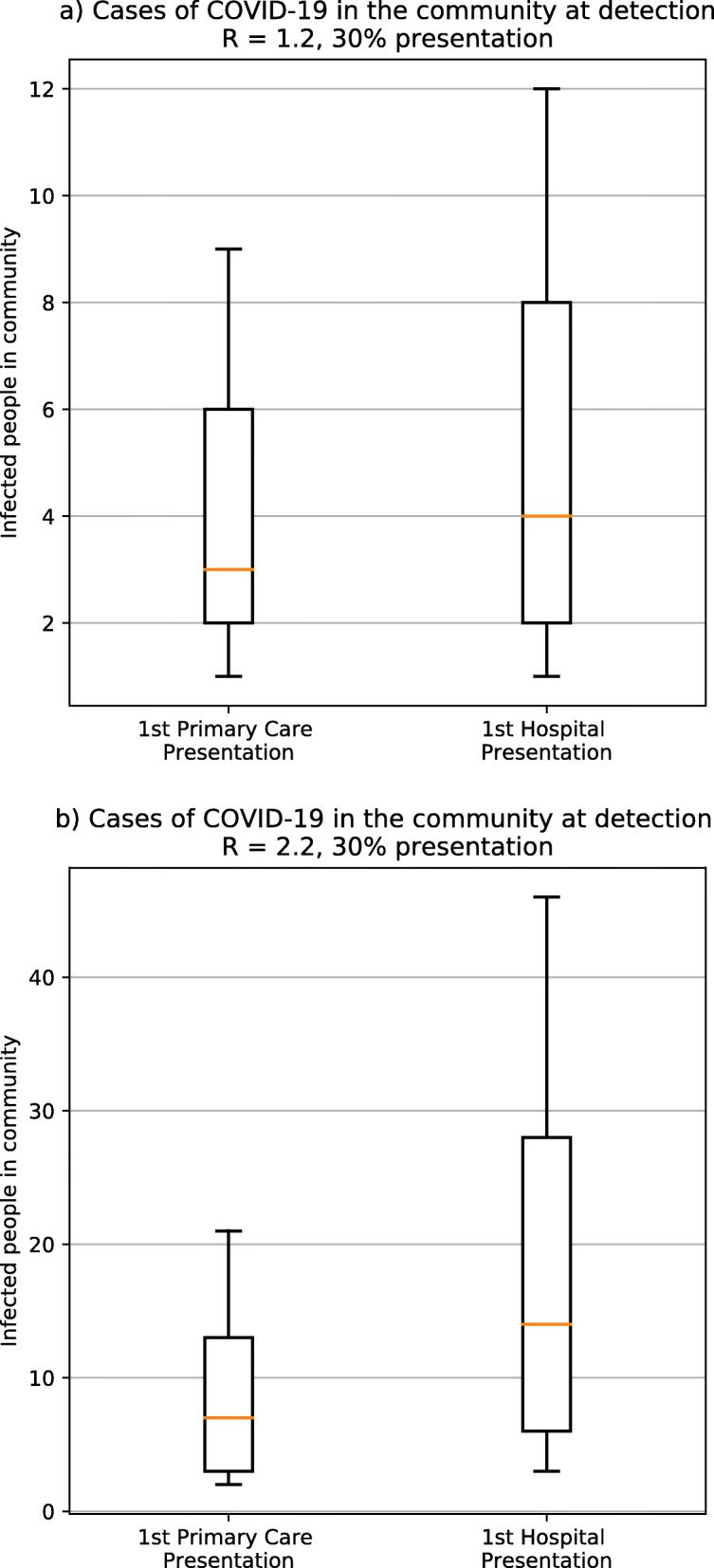
Cases of COVID-19 in the community at detection. Number of infected people in the community when one case is detected in primary care (left) or at hospital (right) for **a** a reproductive number of 1.2 and **b** a reproductive number of 2.2, assuming 30%  of patients with symptoms present to primary care. For each scenario, the box shows the median, 25% and 75% of the distribution of number of cases, while the interval indicates the 10% and 90% limits

Under the default transmission scenario (reproductive number of 2.2), 20% asymptomatic disease and with 30% of all symptomatic cases presenting for testing, there are a median of 14 infected people in the community (8 already infectious) when a severe case with pneumonia is detected at the hospital compared to a median of 7 infected people in the community (4 already infectious) when there is a detection through community /primary care surveillance (Fig. 1). Under the low-transmission scenario (reproductive number of 1.2), there are a median of 4 infected people in the community when a severe case is detected at the hospital compared to a median of 3 infected people in the community when there is a detection is in the community. If 50% of cases are asymptomatic, there are 10 infected people to be traced under default transmission and 4 infected people under low transmission under primary care surveillance. If 50% of cases are asymptomatic and 50% of symptomatic cases present for testing, there are a median of 7 infected people in the community when there is a detection through primary care at high transmission, and a median of 3 infected people in the community at low transmission.

### Efficiency of testing

#### Testing of those with respiratory symptoms in the community

Table 1 assesses, for varied weekly cumulative incidence of symptomatic COVID-19 disease in the community, the estimated number of community cases missed by surveillance-related testing, given varied levels of non-COVID-19 acute respiratory symptoms in the community, and varied testing capacity/uptake. Given a 4% cumulative incidence of acute respiratory symptoms in the population, exhaustive testing of all symptomatic patients would be possible with 60,000 samples per million population per week (Table 1) given COVID-19 rates of up to 0.5%.

**Table 1:** Number of COVID-19 community cases not detected per week under varied prevalence of COVID-19 and non-COVID-19 acute respiratory illness/fever and cough symptom weekly point prevalence, per million population

Weekly point prevalence of non-COVID-19 ARI /fever and cough	Total non- COVID-19 ARI/ fever and cough presentations per week	Number of patients tested per week	0.01% COVID-19 prevalence (100 cases in total)		0.1% COVID-19 prevalence (1000 cases in total)		0.5% COVID-19 prevalence (5000 cases in total)	
			% all (COVID-19 and non-COVID-19) ARI/fever and cough tested	Number of COVID-19 ARI/fever and cough cases in population not tested	% all (COVID-19 and non-COVID-19) ARI/fever and cough tested	Number of COVID-19 ARI/fever and cough cases in population not tested	% all (COVID-19 and non-COVID-19) ARI/fever and cough tested	Number of COVID-19 ARI/fever and cough cases in population not tested
2%	20,000	15,000	75%	25	71%	286	60%	2000
4%	40,000	15,000	37%	63	37%	634	33%	3333
6%	60,000	15,000	25%	75	25%	754	23%	3846
2%	20,000	20,000	100%	0	95%	48	80%	1000
4%	40,000	20,000	50%	50	49%	512	44%	2778
6%	60,000	20,000	33%	67	33%	672	31%	3462
2%	20,000	25,000	124%	0	119%	0	100%	0
4%	40,000	25,000	62%	38	61%	390	56%	2222
6%	60,000	25,000	42%	58	41%	590	38%	3077
2%	20,000	30,000	149%	0	143%	0	120%	0
4%	40,000	30,000	75%	25	73%	268	67%	1667
6%	60,000	30,000	50%	50	49%	508	46%	2692
2%	20,000	40,000	199%	0	190%	0	160%	0
4%	40,000	40,000	100%	0	98%	24	89%	556
6%	60,000	40,000	67%	33	66%	344	62%	1923
2%	20,000	60,000	299%	0	286%	0	240%	0
4%	40,000	60,000	150%	0	146%	0	133%	0
6%	60,000	60,000	100%	0	98%	16	92%	385
2%	20,000	70,000	348%	0	333%	0	280%	0
4%	40,000	70,000	175%	0	171%	0	156%	0
6%	60,000	70,000	116%	0	115%	0	108%	0

Table 2 gives results for testing of 10,000 individuals, under a range of scenarios of varied prevalence of COVID-19 in those being tested and test sensitivity. If we assume a 4% weekly cumulative incidence of symptoms, then for a population of 1 million (i.e. 40,000 individuals with symptoms per week), this would mean multiplying the results in Table 2 by a factor of ((1million × 0.04)/10,000), i.e. by 4, to obtain the number of tests required per million population per week to test all those in the population who are symptomatic. If pooling were used and prevalence of COVID-19 in those symptomatic individuals who were then tested was between 0.05 and 3%, population testing could be achieved with 10,000–15,000 tests per million population by pooling in batches of 4 and with 3000–15,000 tests per million population by pooling of samples in batches of 16 (Table 2). As Table 2 demonstrates, pooled testing is more efficient at detecting disease under all scenarios examined, including when pooling results in a drop in sensitivity to 70%, due to the larger number of individuals that can be tested. Pooling of 1/16 becomes less efficient than 1/4 pooling when prevalence in the tested population exceeds 1%.

**Table 2:** COVID-19 infections missed per 10,000 population tested, with and without pooling, under varied testing capacity, pooling and test sensitivity

Row	Prevalence of COVID-19 in those being tested	Number with disease per 10,000	Sensitivity of pooled test	Exhaustive testing of 10,000 individuals using 1/4 pooling		Exhaustive testing of 10,000 individuals using 1/16 pooling		Percentage cases missed with no pooling (test sensitivity of 99.9%)	
				Col 1	Col 2	Col 3	Col 4	Same number of tests used and individuals tested as in column 1	Same number of tests used and individuals tested as in column 3
				Total tests needed	Percentage of cases missed	Total tests needed	Percentage of cases missed		
1	0.05%	5	99.90%	2530	0%	715	1%	75%	93%
2	0.05%	5	95.00%	2529	5%	711	5%	75%	93%
3	0.05%	5	90.00%	2528	10%	707	10%	75%	93%
4	0.05%	5	80.00%	2526	20%	699	20%	75%	93%
5	0.05%	5	70.00%	2524	30%	691	30%	75%	93%
6	0.10%	10	99.90%	2550	0%	793	1%	75%	92%
7	0.10%	10	95.00%	2548	5%	786	6%	75%	92%
8	0.10%	10	90.00%	2546	10%	778	11%	75%	92%
9	0.10%	10	80.00%	2542	20%	762	21%	75%	92%
10	0.10%	10	70.00%	2538	30%	746	31%	75%	93%
11	1.00%	100	99.90%	2903	2%	2117	7%	71%	79%
12	1.00%	100	95.00%	2884	7%	2045	12%	71%	80%
13	1.00%	100	90.00%	2864	11%	1970	17%	71%	80%
14	1.00%	100	80.00%	2825	21%	1822	26%	72%	82%
15	1.00%	100	70.00%	2785	31%	1673	35%	72%	83%
16	3.00%	300	99.90%	3655	5%	4485	20%	63%	55%
17	3.00%	300	95.00%	3599	9%	4296	24%	64%	57%
18	3.00%	300	90.00%	3541	14%	4103	28%	65%	59%
19	3.00%	300	80.00%	3427	24%	3717	36%	66%	63%
20	3.00%	300	70.00%	3312	33%	3331	44%	67%	67%

Test specificity 99.9% for all scenarios

The above results are primarily influenced by the weekly cumulative incidence of non-COVID-19 acute respiratory illness symptoms when the weekly incidence of COVID-19 symptoms is less than 0.1%. Even at higher incidence of COVID-19, the overall conclusions remain the same (Tables 1 and 2). The main impact of varied community levels of COVID-19 is on absolute numbers of cases missed, and on efficiency gains through pooling of tests. However, when all symptomatic patients present and testing is exhaustive, the number of symptomatic COVID-19 community cases missed by screening remains at 0, whatever the COVID-19 incidence.

#### Testing of asymptomatic groups with high-risk contacts

Table 3 assesses the estimated the number of community cases of COVID-19 missed by asymptomatic screening, given varied testing and Severe Acute Respiratory Syndrome Coronavirus 2 (SARS-CoV-2) asymptomatic infection levels in the population being assessed. For all scenarios, the majority of cases are missed through random asymptomatic screening, whatever the incidence of COVID-19.

**Table 3:** Number of asymptomatic COVID-19 infections per million population not detected under varied prevalence of asymptomatic infection and testing levels

Prevalence of asymptomatic COVID-19 infection	Number of tests conducted	Upper 95% confidence limit of prevalence estimate for COVID-19 in asymptomatic population	Number of cases in screened population	Number of COVID-19 asymptomatic infections missed by screening	% of asymptomatic COVID-19 infections in populations that are missed during screening
0.79%	10,000	0.98%	7900	7821	99.0%
0.48%	10,000	0.64%	4800	4752	99.0%
0.24%	10,000	0.36%	2400	2376	99.0%
0.16%	10,000	0.26%	1600	1584	99.0%
0.10%	10,000	0.18%	1000	990	99.0%
0.79%	15,000	0.95%	7900	7782	98.5%
0.48%	15,000	0.60%	4800	4728	98.5%
0.24%	15,000	0.33%	2400	2364	98.5%
0.16%	15,000	0.24%	1600	1576	98.5%
0.10%	15,000	0.16%	1000	985	98.5%
0.79%	20,000	0.92%	7900	7742	98.0%
0.48%	20,000	0.59%	4800	4704	98.0%
0.24%	20,000	0.32%	2400	2352	98.0%
0.16%	20,000	0.23%	1600	1568	98.0%
0.10%	20,000	0.15%	1000	980	98.0%
0.79%	25,000	0.91%	7900	7703	97.5%
0.48%	25,000	0.57%	4800	4680	97.5%
0.24%	25,000	0.31%	2400	2340	97.5%
0.16%	25,000	0.22%	1600	1560	97.5%
0.10%	25,000	0.15%	1000	975	97.5%

### Test performance and costs versus benefits of pooling

Tables 2 and 4 present test performance for COVID-19 prevalence in the tested population of 0.05–3%, and a range of test characteristics (sensitivity 70–99.9%, specificity 70–99.9%). As Table 2 demonstrates, changes in sensitivity do not have a marked effect on false negatives at low prevalence. At a prevalence of disease of 0.05%, a sensitivity of 90% equates to 0.5 patients missed in screening per 10,000 compared with 0 missed when sensitivity is 99.9% (10% more cases are missed). At a prevalence of 1%, this translates to 11 patients missed in screening compared to 2 missed with a sensitivity of 99.9% (9% more patients are missed). However, as Table 2 also demonstrates, even with a higher false negative rate in a test or pooling strategy, such a test/strategy will be more effective if it allows a substantially greater proportion of the population to be tested. In situations of very limited availability of the polymerase chain reaction (PCR) gold standard testing therefore, testing using a lower-performing but much more readily available testing intervention may be warranted.

**Table 4:** Test performance and number of COVID-19 false positives per 10,000 population tested, for varied test specificities and disease prevalence (given a sensitivity of 99.9%)

Prevalence	Specificity	PPV	False positive rate	Number with disease in population per 10,000 tested	Number of false positives per 10,000 tested
0.0005	0.999	33.32%	0.1%	5	10
0.0005	0.95	0.99%	5.0%	5	500
0.0005	0.9	0.50%	10.0%	5	1000
0.0005	0.8	0.25%	20.0%	5	2000
0.0005	0.7	0.17%	30.0%	5	3000
0.001	0.999	50.00%	0.1%	10	10
0.001	0.95	1.96%	5.0%	10	500
0.001	0.9	0.99%	10.0%	10	1000
0.001	0.8	0.50%	20.0%	10	2000
0.001	0.7	0.33%	30.0%	10	3000
0.01	0.999	90.98%	0.1%	100	10
0.01	0.95	16.79%	5.0%	100	500
0.01	0.9	9.17%	10.0%	100	1000
0.01	0.8	4.80%	20.0%	100	2000
0.01	0.7	3.25%	30.0%	100	3000
0.03	0.999	96.86%	0.1%	300	10
0.03	0.95	38.19%	5.0%	300	500
0.03	0.9	23.60%	10.0%	300	1000
0.03	0.8	13.38%	20.0%	300	2000
0.03	0.7	9.34%	30.0%	300	3000

In regard to positive predictive value, Table 4 demonstrates that it is only with very high specificity (≥ 99.9%), and relatively high prevalence (≥ 1%) that most positive tests are true positives. In all other scenarios, most positives are false positives.

### Epidemiological investigation and contact tracing

**Fig. 2:**
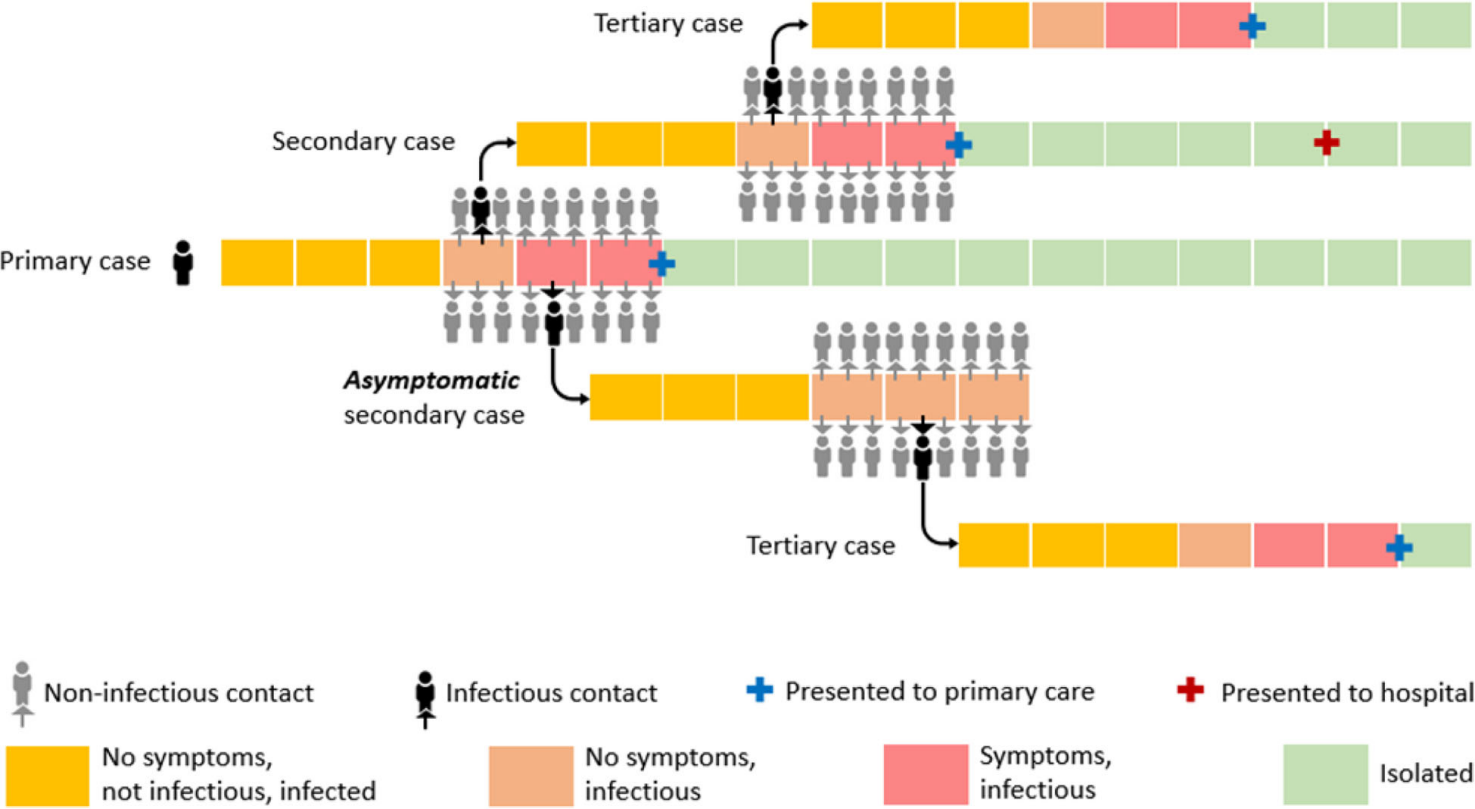
Transmission tree showing detection at primary care or hospital with infectious and non-infectious contacts

For enhanced surveillance to lead to pandemic control, all confirmed cases must be investigated. As Fig. 2 demonstrates, contact tracing must include the following two groups:

- *Upstream contacts* (i.e. the potential source of transmission to the identified case): intensive case finding and testing of these cases, whether symptomatic or not, with PCR-based testing to identify current infection, and with serology to identify past infection. The model indicates 2 or 3 upstream cases (including the primary case) once it is detected at primary care (Figs. 1 and 2). In the absence of social distancing measures, it has been estimated from past outbreaks of other infectious respiratory diseases that each case, on average, has 11 contacts while infectious [32]. Full contact tracing of the entire transmission tree would thus require around 22–33 contacts traced if there are 11 contacts, on average, per case.
- *Downstream contacts* (i.e. those likely to have been infected by the case): optimal follow-up includes intensive case finding and quarantining of these contacts, with home monitoring (such as twice daily temperature checks, GPS monitoring of compliance) and PCR testing for active disease once the 14-day quarantine and follow-up period concludes to ensure they do not have asymptomatic disease. As above, 11 such contacts per case needing 14 days follow-up would be expected [32].

### Indicators of success: monitoring and evaluation of surveillance system performance

#### System coverage, uptake, and completeness

The key indicator of system sensitivity is the proportion of acute respiratory symptoms in the community tested for SARS-CoV-2. This is the primary indicator to be monitored on an ongoing basis, regardless of whether the response aims to supress transmission, achieve elimination, or maintain elimination. As our results indicate, feasible early detection and effective control is reliant on those with symptoms presenting for early testing in the community. The higher this proportion, the less disease there will be in the community at the point of detection, therefore we propose this indicator, with a target of 100% as the key indicator of a sensitive surveillance system with adequate coverage.

Weekly population screening targets (i.e. estimates of the denominator for the above indicator) for all populations under surveillance should be based on Table 1, with the denominator for the target percentage being the estimated levels of acute respiratory symptoms in the community under surveillance. Incidence of symptoms will vary; for example, they are likely increase as social distancing measures are lifted, especially if measures are relaxed during winter. Surveillance through voluntary reporting by community participants, or sentinel surveillance in health services [33], is commonly used to track influenza levels; however, both these strategies are prone to a range of biases including self-selection of volunteers and differentials in health care access and uptake. Surveillance system performance should therefore be assessed and validated through regular, random, community-based surveys of acute respiratory symptom incidence. Such surveys can also monitor community understanding of testing criteria, attitudes towards uptake of testing and related behaviours, feasibility and burden, and support services for enabling testing, as well as other control measures such as quarantine, isolation, mask use and vaccination. The aim is for high levels of understanding, feasibility and support with manageable burden. Such surveys have been demonstrated as feasible on a weekly basis in, for example, an Australian state which has conducted a community-based, random, representative survey each week since April 2020 covering these factors [34]. This weekly survey of ~ 400 participants provides data on the general population that is precise enough to make inferences around change each week, and inferences related to changes over 2–3 months for specific population subgroups of interest.

The following indicators proposed are those that measure the absence of community transmission and/or risk factors for community transmission (i.e. undetected cases in the community, indicated by detected cases with an unknown source of transmission). This is based on the information presented in Fig. 2, which illustrates COVID-19 transmission chains. All of the below indicators measure the absence of community transmission and/or risk factors for community transmission:

- The proportion of those with respiratory symptoms who present for testing on the day of symptom onset.
- The proportion of those with respiratory symptoms who isolate from time of symptom onset to receipt of a negative test result.
- Newly reported cases that are tested on the day of symptom onset (target = 100%).
- The proportion of newly detected cases that have been under quarantine from the time of the exposure event (target = 100%).
- The proportion of age-specific hospitalised new cases and/or deaths relative to new community-acquired cases in the respective age group (target = 0%) [35].
- The proportion of cases with complete follow-up of contacts (target = 100%).
- The proportion of cases with unknown source of transmission (community transmission), with a target of 0%.
- The proportion of newly reported cases that are importations (i.e. acquired disease outside the surveillance area) and/or known contacts of confirmed cases (target = 100%). This is the mirror of the first indicator (i.e. if all detected cases are in contact of cases or imported cases in travellers, then cases with unknown transmission will be 0%).

The number of upstream contacts per case should be at least twice the number of downstream contacts (Fig. 1), and total number of contacts per case is expected to be > 35, unless there is a clear reason for low numbers of contacts. However, numbers of contacts per case can also be much higher [36], as super-spreading events demonstrate. Expected numbers of contacts per case should be reviewed regularly based on population mobility and the characteristics of each case's social network, and contact case definitions updated regularily based on comprehensive testing data from contacts of identified cases, including in high-risk settings. Complete follow-up for upstream contacts should consist of PCR and serological testing (to identify past infection) at time of case detection, while complete follow-up for downstream contacts consists of documented quarantining for 14 days following the last contact with the case, and PCR testing at end of the quarantine period to exclude asymptomatic viral shedding.

#### Progress towards elimination of community transmission

The indicator of successful elimination of community transmission, given the above system coverage, uptake and completeness indicators have all been met, is that the proportion of new cases that are classified as unknown source of infection (community transmission) is 0% over a 28-day period. This is the definition proposed by a range of experts in Australia and New Zealand, based on a period of 2 incubation periods free of disease following known community transmission, and has also been applied to decisions in Australia around the lifting of internal border controls [37].

## Discussion

This study shows that timely detection and management of community transmission of COVID-19 can be achieved with between 3000 and 15,000 tests per million population with pooling depending on the incidence of non-COVID-19 and COVID-19-related respiratory symptoms, and the capacity to trace, on average approximately 33–44 primary contacts per case of community transmission. Testing patients with acute respiratory symptoms is an efficient, effective and feasible strategy for the detection and elimination of transmission chains. The strategy we propose in this paper for COVID-19 surveillance, an earlier version of which was available in preprint form [38], has been integral to Australia’s pandemic response since April 2020 [39, 40].

The most important determinant of the effectiveness of the surveillance system proposed in this paper is uptake of testing in those with respiratory symptoms in the general community. Uptake depends on awareness of and availability and ease of access to testing, community perceptions related to the testing itself and the public health response measures linked to testing such as quarantine and isolation. Ensuring communities understand the need and value of presenting for testing and are supported to access it, is a fundamental requirement for successfully controlling and maintaining elimination of COVID-19. This has been demonstrated in countries that have high testing and contact tracing levels and so have been able to manage outbreaks occuring after the lifting of containment measures [41, 42] and in countries that have successfully controlled transmission through containment but have limited testing, which are seeing resurgence of community transmission [43]. This syndromic surveillance strategy provides a simple and clear message to the community: isolate and get tested if you have respiratory symptoms. Provision of paid sick leave supports compliance with testing and home isolation [1, 44], while strategies for effective community engagement from other pandemics provide guidance to increase the acceptability of this approach [7, 14]. Implementing and evaluating interventions to strengthen community engagement and uptake of COVID-19 response measures, and scaling up those shown to improve participation in the surveillance system, is the most important strategy for achieving effective enhanced surveillance. Countries such as Australia have demonstrated high uptake of burdensome containment measures [4], and of testing [45]. However, although testing rates are amongst the highest in the world, the majority of those with respiratory symptoms are still not being tested [46]. The most common reasons given for this was they do not think they have COVID-19 and that their symptoms were too mild to get tested. It is reasonable to expect that testing uptake could be further improved with a consistent and coordinated community engagement strategy.

Promising strategies for further enhancing testing coverage include self-collection of samples [47]. Influenza studies have found that self-collection reduced sensitivity (87% sensitivity compared to clinician collected samples) [48]. As with pooling, self-collection may still result in greater detection, despite the reduction in sensitivity, if it increases the number of tests carried out. In both cases, messaging to the public must indicate the possibility of false negatives and that those with symptoms should isolate regardless of test results. Another promising strategy under review is testing of saliva samples, which will both allow self-collection and not require equipment such as swabs, another limit on testing capacity [49].

Surveillance through hospital presentations with pneumonia has low sensitivity, particularly as the reproductive number increases, and would be weaker still if measures to protect vulnerable groups [23] such as the elderly, immunocompromised or those with chronic respiratory or cardiovascular disease [50, 51] are successful, thus reducing the proportion of cases with severe disease [23]. The gains from community testing compared with hospital surveillance increase as the reproductive number increases, meaning community-based surveillance strategies will increase in comparative effectiveness as restrictions on social movement and interaction are lifted. Random testing of sentinel asymptomatic groups is inefficient at most levels of likely prevalence. If asymptomatic testing were intended to detect cases, it would need to be repeated to constitute an effective surveillance system, entailing a high level of frequent, repeated and large-scale testing unlikely to be feasible on an ongoing basis in most settings [52]. Potential false negatives when testing asymptomatic people further decreases the value of this approach. An exception may be very clearly defined, small population subgroups where, once transmission in the general community has been controlled, the risk of reintroduction from higher prevalence settings and spread to the general community is high. An example of this is staff in COVID-19 quarantine facilities. Subsequent to the initial control of transmission, all major outbreaks in Australia requiring population-level restrictions on movement and social interaction have been seeded by breaches in quarantine facilities housing or transporting international travellers returning to Australia from higher prevalence countries [53–55]. In such settings, frequent testing of staff is feasible and has high value in terms of preventing reintroduction, even if yield per test is low.

Where prevalence is low and there is limited testing capacity, pooling considerably increases coverage. Even with a decrease in sensitivity of pooled tests, the number of false negatives is small compared to the number that is untested. Pooling can also greatly reduce the need for testing reagents.

Evidence suggests that sensitivity of viral PCR is high, at least 95%, although study findings are heterogeneous [56]. Table 2 demonstrates that reduced test sensitivity results in higher false negatives rates when prevalence is higher than 0.01%. Reduced test sensitivity impacts surveillance effectiveness similarly to reduced testing uptake, although the magnitude of effect is likely considerably less than that of reduced uptake (e.g. 70% of those with respiratory symptoms are not being tested currently in Australia) [28]. At low prevalence, small reductions in specificity considerably reduce the positive predictive value, and most positive tests are false positives. Although specificity is considered high (100%) for PCR tests, the impact of reduced specificity is an important consideration when assessing rapid diagnostic tests coming to market.

Upstream contact tracing, including widespread testing of asymptomatic contacts of cases, is the main situation where testing of asymptomatic cases is warranted. Currently, some cases identified in Australia are classified as ‘unknown’ source of exposure [57]. As Fig. 2 demonstrates, this means that their source of exposure may have initiated multiple chains of transmission. Widespread testing of all contacts, upstream and downstream, including low-risk contacts around such cases, is the most effective strategy for filling these gaps in transmission mapping. Although investigation and quarantining of downstream contacts is part of most surveillance strategies, follow-up of upstream contacts is not uniformly undertaken, but is a feature of those settings implementing successful control [1, 42, 58, 59]. Investigation of contacts can be supported through training and integration of community volunteer networks into existing surveillance and contact tracing teams. Software may assist considerably in these efforts [19] and, linked to measures to increase uptake and adherence, is again an important part of follow-up in those settings with effective control [60].

With increased testing capacity, inclusion of symptoms emerging as useful predictors of early and/or mild disease (e.g. anosmia and loss of taste) [61] may increase the sensitivity of the system and allow earlier detection of transmission chains, especially once containment is lifted and the reproductive number increases accordingly. Screening of wastewater for evidence of community transmission is receiving increased attention [62] and could provide a useful addition to primary care screening systems. If sufficiently sensitive, it could also be used in ongoing monitoring of high-risk facilities such as hospitals and aged care facilities to identify early transmission. As it cannot identify infected individuals of chains of individuals, any wastewater testing must be linked to intensive human surveillance to effectively support control of COVID-19 community transmission.

The determinant of the number of individuals requiring testing is the weekly cumulative incidence of respiratory symptoms in the general community. If COVID-19 has a seasonal pattern as with similar SARS-CoV viruses [63], transmission during winter is likely to be amplified. Strong containment and surveillance aimed at elimination of COVID-19 before the peak of winter will optimise the value of this investment.

We found that early identification, isolation and testing of symptomatic individuals linked to effective contact tracing of their social networks is critical, and this is supported by previous modelling of these factors [64]. However, although average contact numbers are useful when transmission is high, once low levels are reached, there can be great variation in contact patterns. Recent outbreaks in Australia have seen relatively few cases resulting in thousands of contacts needing to be managed [65]. Novel strategies such as pre-emptive quarantining of the contacts of contacts, as well as the emergence of more transmissible strains [66], further emphasise the urgent need for empirical data to define operational requirements to implement these measures in practice.

Our study only assesses surveillance and control measures for local transmission. A key contributor to transmission in Australia has been the management of Australia’s borders, both air and sea. Hotel quarantining of new international arrivals began on March 28th 2020 [67] and, coupled with restrictions on movement and social interaction, contributed to a marked reduction in locally acquired disease. An enhanced surveillance system that has achieved elimination of detectable disease is designed to detect and manage early re-introductions or emergence, including travel-related disease. However, continuation of effective border controls between areas with ongoing community transmission and those without, once other social distancing and control measures are lifted, will reduce the pressure on the surveillance system to identify and manage imported disease. The other major challenge to the surveillance system will come from mass amplification events. Both during the current COVID-19 pandemic, and in past, significant outbreaks of other infectious diseases, such events centre around religious or burial activities [68], other large gatherings where contact occurs [69], specific settings where aerosol generating activities and close proximity are involved such as choirs and gyms, and around health facilities and other institutional settings [70]. Limiting the opportunities for transmission in such settings will be crucial to ensuring that single super-spreading events that overwhelm contact tracing system do not occur.

## Conclusions

Given our findings, we recommend exhaustive testing of patients with respiratory symptoms in the community as the most efficient and feasible means of detecting community transmission of COVID-19 under conditions of both restricted and close to normal social interaction. Once community cases are identified, detailed and meticulous upstream and downstream contact tracing, linked to quarantining of all contacts, and both antigen and serological testing of upstream contacts who may be the source of infection, will support elimination of community transmission, and rapid control if and when reintroductions of disease occur. This strategy optimises the likelihood of remaining in the elimination phase while allowing for ongoing lifting of containment measures. Community engagement is critical for successful implementation of this strategy to ensure high levels of testing uptake and compliance with follow-up measures in identified cases and their contacts.

## Supplementary Information

**Additional File 1.** Model code.

## Data Availability

All data and relevant methods of analysis are described in the paper and supplementary materials and can be replicated on this basis. No other primary data were utilised.

## Abbreviations

COVID-19: Coronavirus Disease 2019
PCR: Polymerase chain reaction
SARS-CoV-2: Severe Acute Respiratory Syndrome Coronavirus 2
