# Supplementary material for: Exit strategies: optimising feasible surveillance for detection, elimination, and ongoing prevention of COVID-19 community transmission
Source: BMC Med. 2021 Feb 17;19:50. doi: 10.1186/s12916-021-01934-5 (PMC7887417; doi:10.1186/s12916-021-01934-5)
Supplement: Supplementary file 1 — Additional File 1. Model code. [file 12916_2021_1934_MOESM1_ESM.docx]

# Supplementary Document: Model code

The following python code specifies the mathematical model used in Lokuge et al “Exit strategies: optimising feasible surveillance for detection, elimination, and ongoing prevention of COVID-19 community transmission”

#!/usr/bin/env python

# coding: utf-8

# # Model for Exit strategies

#

# This notebook contains the model used in the article *Exit strategies: optimising feasible surveillance for detection, elimination, and ongoing prevention of COVID-19 community transmission*

# ## Import libraries

# In[28]:

import numpy as np

import matplotlib.pyplot as plt

# ## Define functions

# There two key functions that predict the number of individuals in each category at each time.

#

# ### runStochastic

#

# This function iterates through one simulation. It keeps progressing one time step at a time until one of the following three conditions have been met:

# * The simulation has reached the time limit of the simulation. (Set to 50 days)

# * There are no longer any infected people

# * There is at least one GP case and at least one hospitalised case

#

# Once it has finished simulating the model the function then captures for both first encounter at GPs and Hospitals:

# * the time taken for the first encounter to occur

# * the number of infected individuals at the time of the first encounter

# * the number of exposed individuals at the time of the first encounter

#

#

# ### stochasticModel

#

# This function defines how the stochastic model progresses infections and time. Key variable names are explained below. The model is a standard stochastic SEIR model. Textbooks such as Keeling and Rohani 2007 will provide a basis to these types of equations.

#

# * *N*: Population

# * *p_s*: Probability that a case has severe symptomatic illness

# * *p_m*: Probability that a case has mild symptomatic illness

# * *p_a*: Probability that a case is asymptomatic

# * *p_gp*: Probability than an individual presents to primary care

# * *beta*: Transmission parameter

# * *sigma1*: Inverse of the latent period

# * *sigma2*: Inverse of the time from onset of infectiousness until symptom onset

# * *gamma*: Inverse of the symptomatic infectious period

# * *tau_s*: Rate at which severe cases present to hospital following the end of the infectious period

#

#

# * *S*: Susceptible

# * *E1*: Infected but not infectious or symptomatic

# * *E2*: Infected, infectious but not symptomatic

# * *I*: Infectious and showing symptoms

# * *V*: Severe case quarantine

# * *H*: Present at Hospital and go into quarantine

# * *G*: Present at GP and go into quarantine

# * *R*: Recovered

# In[22]:

def runStochastic(param,y0):

T = param[1]

timespan = np.arange(T) # Time limit of simulation

yval = np.copy(y0) # Initial distribution of disease in population

tval = 0

t = np.array(tval)

y = np.matrix(yval)

while ((tval < T) and (y[-1,1]+y[-1,2]+y[-1,3]>0) and (y[-1,5]*y[-1,6]==0)):

# run while within time limit, people are infected and there aren't both hospitalised AND GP cases yet

[tval,yval] = stochasticModel(tval,yval,param)

t = np.append(t,tval)

y = np.append(y,[yval],axis=0)

iHall = np.where(y[:,5]!=0)[0] # times where there is a hospitalised case [i]nfected[H]ospital[all]

if(len(iHall)>0):

indH = iHall[0] # index

tH = t[indH] # time of hospitalisation

eH = y[indH,1] # number of exposed cases at first hospitalisation

iH = y[indH,2]+y[indH,3] # number of infectious cases at first hospitalisation

else:

tH = 2*T

eH = 0

iH = 0

iGall = np.where(y[:,6]!=0)[0] # times where there is a case presenting to GP [i]nfected[G]P[all]

if(len(iGall)>0):

indG = iGall[0]

tG = t[indG]

eG = y[indG,1]

iG = y[indG,2]+y[indG,3]

else:

tG = 2*T

eG = 0

iG = 0

return tH, eH, iH, tG, eG, iG

# In[23]:

def stochasticModel(t,y0,param):

N=param[0]

p_s = param[2]

p_m = param[3]

p_a = param[4]

p_gp = param[5]

beta = param[6]

sigma1 = param[7]

sigma2 = param[8]

gamma = param[9]

tau_s = param[10]

y=np.copy(y0)

S = y[0] # Susceptible

E1 = y[1] # Infected but not infectious or symptomatic

E2 = y[2] # Infected, infectious but not symptomatic

I = y[3] # Infectious and showing symptoms

V = y[4] # Severe case quarantine

H = y[5] # Present at Hospital and go into quarantine

G = y[6] # Present at GP and go into quarantine

R = y[7] # Recovered

r = np.zeros(7)

r[0] = beta*S*(I+E2)/N # S - E1

r[1] = sigma1*E1 # E1 - E2

r[2] = sigma2*E2 # E2 - I

r[3] = gamma*I*(p_m*(1-p_gp) + p_a) # I - R

r[4] = gamma*I*p_m*p_gp # I - G

r[5] = gamma*I*p_s # I - V

r[6] = tau_s*V # V - H

rtot = np.sum(r)

newy = y

if rtot < 0.001:

newt = 100

else:

rn2 = np.random.ranf()

i = 1

while i<7 and rn2 > np.sum(r[0:i])/rtot:

i = i+1

if i == 1: #S -> E1

newy[0] = newy[0] - 1

newy[1] = newy[1] + 1

elif i == 2: # E1 -> E2

newy[1] = newy[1] - 1

newy[2] = newy[2] + 1

elif i == 3: #E2 -> I

newy[2] = newy[2] - 1

newy[3] = newy[3] + 1

elif i == 4: #I -> R

newy[3] = newy[3] - 1

newy[7] = newy[7] + 1

elif i == 5: #I -> G

newy[3] = newy[3] - 1

newy[6] = newy[6] + 1

elif i == 6: #I -> V

newy[3] = newy[3] - 1

newy[4] = newy[4] + 1

if np.random.ranf() < p_gp:

newy[6] = newy[6] + 1 # this is an additional GP presentation of a severe case who is counted in both G and [later] in H

elif i == 7: #V -> H

newy[4] = newy[4] - 1

newy[5] = newy[5] + 1

rn1 = np.random.ranf()

newt = t - np.log(rn1)/rtot

return newt,newy

# In[36]:

def printstats(g,h,alpha):

r = [1.2,2.2,2.6]

for i in range(len(r)):

gnew = np.sort(g[i])

hnew = np.sort(h[i])

gps = [gnew[round(len(gnew)*alpha/2)],gnew[round(len(gnew)*0.25)], np.median(gnew),gnew[round(len(gnew)*0.75)],gnew[round(len(gnew)*(1-alpha/2))]]

hospitals = [hnew[round(len(hnew)*alpha/2)],hnew[round(len(hnew)*0.25)],np.median(hnew),hnew[round(len(hnew)*0.75)],hnew[round(len(hnew)*(1-alpha/2))]]

print('R = '+ str(r[i]))

print('GP: '+ str(gps))

print('Hospitals: '+ str(hospitals))

print('')

# ## Run Simulation

# In[42]:

runs = 5000 # number of stochastic runs

N=397000 # population size

T=50 # time to run model for

Rnaught = 2.2 # reproduction number

p_s = 0.2 # probability infected has severe symptomatic illness

p_m = 0.6 # probability infected is has mild symptomatic illness

p_a = 0.2 # probability infected is asymptomatic

p_gp = 0.5 # probability mild infected will present to primary care

sigma1 = 1/3 # inverse of latent period

sigma2 = 1/1 # inverse of time from onset of infection to onset of symptoms if symptomatic

gamma = 1/2 # inverse of infectious period following onset of symptoms

tau_s = 1/5 # rate at which severe case presents to hospital following end of infectious period

beta = Rnaught/(1/sigma2 + 1/gamma) # transmission rate

param = [N, T, p_s, p_m, p_a, p_gp, beta, sigma1, sigma2, gamma, tau_s]

# Initial conditions

E1s=1 #Assume one initial exposed case

Ss=N-E1s # Make the rest susceptible

E2s=0

Is = 0

Vs = 0

Hs=0

Gs=0

Rs=0

y0s=[Ss, E1s, E2s, Is, Vs, Hs, Gs, Rs]

tH = np.zeros((3,runs))

eH = np.zeros((3,runs))

iH = np.zeros((3,runs))

tG = np.zeros((3,runs))

eG = np.zeros((3,runs))

iG = np.zeros((3,runs))

RList = [1.2, 2.2, 2.6]

for i in range(len(RList)):

beta = RList[i]/(1/sigma2 + 1/gamma)

param = [N, T, p_s, p_m, p_a, p_gp, beta, sigma1, sigma2, gamma, tau_s]

j = 0

while j < runs:

tH[i][j], eH[i][j], iH[i][j], tG[i][j], eG[i][j], iG[i][j] = runStochastic(param,y0s)

if (tH[i][j] < T) and (tG[i][j] < T):

j = j+1

# if j%500==0:

# print(j)

all_E_H = eH + iH

all_E_G = eG + iG

printstats(all_E_G, all_E_H, 0.2)

# ## Plot from results

# In[51]:

label = ['1st Primary Care \nPresentation', '1st Hospital \nPresentation']

d_r12 = [all_E_G[0],all_E_H[0]]

d_r22 = [all_E_G[1],all_E_H[1]]

d_r26 = [all_E_G[2],all_E_H[2]]

plt.rc('font', size=10)

presentation = '30%'

fig, ax = plt.subplots(nrows=3, ncols=1, figsize=(6, 20))

ax[0].set_title(' a) Cases of COVID-19 in the community at detection \nR = 1.2, {} presentation'.format(presentation))

ax[0].boxplot(d_r12, whis=[10,90], showfliers = False, patch_artist = False, labels=label)

ax[0].set_ylabel('Infected people in community')

ax[1].set_title('b) Cases of COVID-19 in the community at detection \nR = 2.2, {} presentation'.format(presentation))

ax[1].boxplot(d_r22, whis=[10,90], showfliers = False, patch_artist = False, labels=label)

ax[1].set_ylabel('Infected people in community')

ax[2].set_title('c) Cases of COVID-19 in the community at detection \nR = 2.6, {} presentation'.format(presentation))

ax[2].boxplot(d_r26, whis=[10,90], showfliers = False, patch_artist = False, labels=label)

ax[2].set_ylabel('Infected people in community')

for a in ax:

a.yaxis.grid(True)

plt.show()
